# Gender (in)equality among employees in elder care: implications for health

**DOI:** 10.1186/1475-9276-11-1

**Published:** 2012-01-04

**Authors:** Sofia Elwér, Lena Aléx, Anne Hammarström

**Affiliations:** 1 Department of Public Health and Clinical Medicine, Umeå University, SE 901 87 Umeå, Sweden; 2 Department of Nursing, Umeå University, SE 901 87 Umeå, Sweden

**Keywords:** Content analysis, focus groups, gender, health experiences, work environment, workplace

## Abstract

**Introduction:**

Gendered practices of working life create gender inequalities through horizontal and vertical gender segregation in work, which may lead to inequalities in health between women and men. Gender equality could therefore be a key element of health equity in working life. Our aim was to analyze what gender (in)equality means for the employees at a woman-dominated workplace and discuss possible implications for health experiences.

**Methods:**

All caregiving staff at two workplaces in elder care within a municipality in the north of Sweden were invited to participate in the study. Forty-five employees participated, 38 women and 7 men. Seven focus group discussions were performed and led by a moderator. Qualitative content analysis was used to analyze the focus groups.

**Results:**

We identified two themes. "Advocating gender equality in principle" showed how gender (in)equality was seen as a structural issue not connected to the individual health experiences. "Justifying inequality with individualism" showed how the caregivers focused on personalities and interests as a justification of gender inequalities in work division. The justification of gender inequality resulted in a gendered work division which may be related to health inequalities between women and men. Gender inequalities in work division were primarily understood in terms of personality and interests and not in terms of gender.

**Conclusion:**

The health experience of the participants was affected by gender (in)equality in terms of a gendered work division. However, the participants did not see the gendered work division as a gender equality issue. Gender perspectives are needed to improve the health of the employees at the workplaces through shifting from individual to structural solutions. A healthy-setting approach considering gender relations is needed to achieve gender equality and fairness in health status between women and men.

## 

Gender equality can be defined as the absence of discrimination in relation to opportunities, allocation of resources or benefits and access to services for women and men [1]. In social sciences the concept of gender equality has been used as the foundation for notions of gender justice [2]. Inequalities, or differences, between women and men have been seen as a product of social power relations and therefore inherently unfair. In a Nordic setting the discourses about the concept of gender equality have been critically studied in the everyday life of families [3] as well as in politics [4]. Attitudes towards gender equality have also been studied in relation to employment opportunities, showing that positive attitudes to equal opportunities are often held by the same people who stress practical obstacles [5]. However, to our knowledge there are no studies investigating how the view of gender equality is related to health experiences. In this paper we focus on how gendered social processes in working life are related to health experiences of employees at a woman-dominated workplace. We use the concept of gender (in)equality to describe these processes. We put the negation of the concept in brackets to enable a simultaneous discussion about the processes of gender equality and inequality.

A central debate related to gender equality concerns sameness and difference. This debate involves different methods for diminishing gendered hierarchies and creating a fair society. A sameness perspective argues that men and women are basically the same and that fairness is created through equal opportunities, abolishing the socially constructed gender differences [6,7]. According to the difference perspective women and men are essentially different and fairness is created through valuing women and men equally [7,8]. A difference perspective on gender equality is dependent on a definition of which differences to accept as essential, whereas a sameness perspective offers a distinct definition, where same is considered as equal. The sameness perspective is considered suitable when social obstacles that prevent fairness in health between women and men are in focus [9]. The gender perspective in this paper is therefore guided by theories of feminist justice, which emphasise the importance of gender equality, as sameness, in paid and unpaid work [6].

The research field of gendered organizations has shown how gendered practices of working life shape hierarchies, jobs, organization cultures and relationships [10,11]. These gendered practices create gender inequalities through horizontal and vertical gender segregation in work, which can lead to inequalities in health between women and men [12,13]. The overall pattern of gender relations within an organization could be analyzed as gender regimes, involving four dimensions of gender relations: labor, power, symbolic relations and emotional relations [14,15]. A local gender regime can differ from the overall social pattern of gender relations which constitute the gender order of society. At each workplace the gender regimes construct our work situation, which is important for health status. Gender (in)equalities at workplaces is of importance for gender-related health [12] and can be related to health in both positive and negative ways. There might also be differences in how gender (in)equalities relate to health for women and men at the workplace. Women might, for example, be protected from heavy lifting in the workplace because men are considered to be stronger. When gender constructions have been studied in relation to health, masculinities and especially hegemonic masculinities have emerged as mainly negative for health because of the risk behaviors that are connected to such a masculinity [16]. Women's health status is generally negatively affected by the discrimination and disadvantage that they experience as they carry out the gendered activities in their lives [17]. Despite this, femininity, or being a woman, has mainly been connected to positive health behaviors, for example utilizing health care more frequently and drinking less alcohol [18]. An understanding of gender regimes in relation to health experiences is crucial in health promotion aimed at ensuring safe and supportive working environments [19].

The Swedish government's gender equality policy states that women and men should have the same power to shape society and their own lives by having the same opportunities, rights and responsibilities [20]. At the workplace this policy is ensured through the Swedish Act Concerning Equality between Men and Women, stating that all employees are required to work actively to ensure gender equality by preventing sex discrimination and promoting gender equality [21]. A working life characterized by diversity, gender equality and nondiscrimination is also a part of the Swedish public health policy [22]. However, the construction and legitimacy of gender equality plans and policies at the workplace are dependent on how gender equality is comprehended and put into action in the workgroup.

In Sweden women participate in the paid labor force to almost the same extent as men, 80 compared to 86 percent, but the labor market is strongly segregated [23]. Only 15 percent of the workers are in a profession where women and men are equally represented to at least 40-60 percent [23]. The gender-segregated labor market indicates that gender plays a major role in work allocation and that work is an arena where gender is constructed [24]. Working conditions in woman-dominated sectors are often characterized by high demands and low control, which has negative health consequences as regards, for example, psychological distress for both women and men working under these conditions [13,14,25,26].

Caring work, both paid and unpaid, is traditionally connected to ideas about femininity as well as to low status, and women also constitute a majority of the professional health care workers [14,27-29]. Joan Tronto [29] distinguishes "caring for" as a traditional sphere of women which involves attentiveness to the needs of others in the shape of commitment of time and effort, perhaps at a high price to oneself. Muscular pain, tiredness and exhaustion have been shown to be overrepresented health problems among employees in elder care [30-32]. Experiences of depreciation and devaluation have been described as part of the work experience of employees in elder care [32-35]. The low status of caring work can also in itself be negative for the health of the caregivers because the perception of one's own position in a social hierarchy is important for self-perceived health [36]. Earlier research has shown that women at woman-dominated workplaces believe that wages and the status of their work would increase if more men were employed [37]. Studies of working conditions and job stress among nurses often focus on what such a situation means for the patients' health, whereas few studies focus on the experiences of the caregivers themselves [38]. The tendency to neglect the working conditions of the caregivers may be an expression of a caring discourse of putting the needs of others before one's own needs [29,39]. By informally taking greater responsibilities and risks when caring for patients, the caregivers protect the patients from shortcomings in the health care system [40]. Although caring work is highly gendered, gender equality is rarely discussed, possibly because the domination of women makes it difficult to compare the working conditions of women and men. Internationally, there are a number of studies evaluating the work stressors of caregivers [41-44] but little attention is paid to gendered aspects of these stressors. Studies of occupational stress tend to have an individual focus that neglects how structural factors such as gender relations can influence individual behavior [35]. In a Swedish context there are a few recent studies of gender in elder care [45,46] but none of them focus on health aspects. To focus on gender equality and health-related issues at womandominated workplaces might be a way forward to identify social obstacles that prevent fairness in health status between women and men. We therefore want to focus on how gender (in)equality is constructed at a woman-dominated workplace in elder care and how this construction is related to health experiences. In this study we regard health experiences as constructed by physical and mental resources in different social contexts [47]. The social context where gender is an important part can both facilitate and complicate an individual's possibilities to experience health, and consequently experiences of health reflect the whole life situation [48]. Our aim is to analyze what gender (in)equality means for the employees at a woman-dominated workplace and to discuss possible implications for health experiences.

## Method

### Design and Setting

We used a qualitative approach to study the employees' views and experiences of gender (in)equality at work and possible implications for health experiences. We conducted the study in two nursing homes for elderly in need of care and medical treatment in a medium-sized Swedish town. The nursing homes had personnel present day and night. The occupational groups employed at the nursing homes were assistant nurses, nurses, occupational therapists, physiotherapists and managers. Assistant nurses constituted the largest occupational group and were the only group which also included men. The other occupational groups included only women. The number of participants from different occupational groups is shown in table 1. The managers were responsible for the assistant nurses while the other occupational groups had their managers situated elsewhere. The study was approved by the regional board of ethical review in Umeå.

**Table 1:** Occupational Groups in Focus Groups

Occupational groups	Number of participants (n = 45)	Women (n = 38)	Men (n = 7)
Assistant nurses	30	23	7
Nurses	9	9	0
Physical therapists	1	1	0
Occupational therapists	2	2	0
Managers	3	3	0

### Participants

The head of geriatric care of the municipality distributed information about the study to all 25 nursing homes and homes for elderly in the city during the spring of 2006. Two workplaces showed an interest in the project. We invited all caring staff and managers at these two workplaces, in total 5 wards and 113 employees (97 women and 16 men) to participate in the study. In total 45 caregivers and managers participated (38 women and 7 men). Written informed consent was obtained from the participants of this study. The participants were also informed verbally that their participation was voluntary, that confidentiality was guaranteed, and that they had the right to leave the study at any time at their discretion.

### Data Collection

We started the project with two meetings where the researchers presented the project. The meetings included a brief introduction about gender equality and workplace-related health to describe the background and aim of the project. The introduction was followed by focus group discussions in two rounds, the second of which is analyzed in this paper. The first round focused on the employees' experiences of work-related health and is described elsewhere [32].

We base this paper on seven focus group discussions which we conducted during the spring of 2007 following focus group research principles [49]. We divided the caregivers into seven groups with participants from different occupations and workplaces. In each group the assistant nurses came from the same workplace whereas the other occupational groups came from the other workplace.

The focus groups consisted of between three and ten participants. Two groups included only women and the other five groups included both men and women. The focus group discussions lasted for 90 minutes and took place at one of the workplaces during paid work time to enable all employees to participate. We chose the time and place for the focus groups to suit the participants as much as possible. A moderator (SE) led the discussions, introducing questions concerning gender equality. The questions followed a thematic question guide that was formulated to provoke discussions in the group. The thematic question guide included the areas 'the meaning of a gender-equal workplace', 'gender equality connected to health and ill health' and 'the importance of gender equality'. When gender equality issues were discussed the moderators followed up with questions on how this might be related to health experiences. Each area was introduced and followed up by the moderator and the assistant moderator (LA) and discussed in the group. The moderator and assistant moderator were actively involved in creating possibilities for all participants to make themselves heard in the group. The focus group discussions were tape-recorded and transcribed verbatim. For ethical reasons all transcribed text was anonymous, thus no information (e.g. the sex or occupation) is available about the individual participants.

### Analysis

We used qualitative content analysis according to Graneheim and Lundman [50] to analyze the transcribed text. Two of the authors (SE and LA) read through the text several times with as open minds as possible in order to grasp the content and look for variations in the text. The text was divided into meaning units which were coded. We discussed the first coding and differences were resolved. The first author used the Open Code computer package [51] for additional systematizing of the codes by dividing them into preliminary categories. The codes and categories were then compared discussed and scrutinized by all the authors, resulting in consensus about six categories. The themes were formulated from the underlying meaning in the meaning units, codes and categories to describe the employees' views and experiences of gender equality and gender inequality at work and its relation to health experiences. The themes were seen as threads running through meaning units, codes and categories. Throughout the process the findings have been discussed back and forth several times. The analysis and findings have also been discussed with other researchers at various seminars and conferences. Examples of meaning units, codes, categories and themes are presented in Table 2.

**Table 2:** Examples of Meaning Units, Codes, Category and Theme

Meaning units:	Codes:	Category:	Theme:
But isn't it that you have a certain predisposition for some things.	Predisposition	Gendered Specialization	Justifying gender inequalities with individualism
There are some [of the elderly], that don't have any relatives, that have ragged clothes and things like that, but all of us can't sew, that's the way it is, and then those who can sew can do it.	Using sewing skills		
Hair is a typical women's job. If we take that.	Women's job		
I mean, of course a man could learn how to do that as well but he can be much better at something else that a woman is much worse at.	Gendered competences		
Then you might as well do a swap of work tasks.	Swap of work tasks		

## Results

The analysis resulted in two themes, "Advocating gender equality in principle" and "Justifying gender inequality with individualism", which will be described below. Themes with categories are presented in Table 3.

**Table 3:** Themes with categories

Themes	Advocating Gender Equality in Principle	Justifying Gender Inequality with Individualism
Categories	Equal Salary	Gendered Specialization
	More Men	Women Taking on Responsibility
	Equal Work Division	Women Managing Family and Health

### Advocating Gender Equality in Principle

This theme was built up of the categories "equal salary", "more men" and "equal work division". Gender equality was mainly described in structural and quantifying terms, as demonstrated by a participant who said: "Well, the dream is to have the same number of men as women [at the workplace], then you're really gender-equal". There were however also expressions of participants with a more relational approach to gender equality. They were prepared to examine their own actions and the gender relations at their workplace. However, the participants showed a higher level of consensus about the ideal of structural gender equality issues and less about the more relational issues where differences between women and men were defended more, as will be discussed in the second theme. The ideal of gender equality was expressed as a matter of justice and fairness and not spontaneously connected to health experiences for the participants.

In the category "equal salary" the participants expressed great agreement in their initial definition of gender equality as the same pay for the same work. One of the participants explained:

"Yes, one would of course like to say that it is an important question [gender equality]. But for me gender equality, what I feel is not that you do the same things, that's not gender equality for me. For me it's more that I should have an equal salary for equal work."

The individually negotiated salaries at the workplace were considered unfair because the negotiations did not favor the best caregivers but rather the most verbal co-workers with the ability to negotiate and co-workers that were taken on when the salary level was high. Participants said that they preferred the former collective system where the salary was based on education and number of years in the profession. They also considered the low salaries of their occupation unfair compared to salaries at man-dominated workplaces.

In the category "more men" the participant wanted to increase the proportion of men in the professions and they stressed that more men at the workplace would increase the salary and status of the profession and make the workplace gender-equal. Mixed groups were described as taking the edge off the gendered language of single-sex groups and creating a positive, more open atmosphere and a better work climate. The participants said that they did not have any direct power to influence the recruitment of men. The participants also found it difficult to judge the gender equality at their own workplace because of the high domination of women (few men to compare with). The workplace was described as a woman's world with women's conditions.

In the category "equal work division" there were different opinions of the extent to which gender equality should be an organizing principle. Some participants firmly defended the idea of gender equality in the division of work tasks and responsibilities. They declared that everyone had to perform all work tasks irrespective of previous knowledge and took as an example that men had to learn how to curl the hair of the old ladies or sew a button on the clothes of the elderly. In the name of fairness, no one should be able to get away with not performing some work tasks. "Just because it's men they should not escape ironing or doing the dishes, it's a lot like that sometimes, that 'they're men - they can't'." The participants' differed in their assessment as to whether gender equality in work tasks and responsibility was practiced at their workplace. Some of the participants felt that less was expected of their male colleagues and that the men in the workgroup got more appreciation, which was considered unfair. To achieve an equal work division the caregivers found it important to have a dialogue at the workplace and discuss how work was divided. One workplace had highly specified work descriptions to ensure that everyone reached the goals.

### Justifying Gender Inequality with Individualism

This theme was built up of the categories "gendered specialization", "women taking on responsibility" and "women managing family and health". The theme included what the participants described as individual differences and what they therefore often saw as acceptable exceptions to the gender equality ideal described in the previous theme. In other words, this concerns how the participants explained and justified gender inequality. The participants did not necessarily see these exceptions as gender inequalities but rather as ways of getting work to run smoothly. The justification of gender inequality was related to health experiences in a variety of ways, as presented in the categories below.

In the category "gendered specialization" the participants said that specialization, whereby everyone took care of the tasks that they were skilled in, was a resource-efficient solution for the division of work both at home and at work. Women were described as doing more laundry, sewing, taking care of flowers, baking and curling the hair of the elderly ladies. Men were described as stronger and more suited to handle heavy lifts, although this seemed to be connected to moving furniture and things, rather than lifting the elderly, which both women and men did.

"Everyone can't do the same things, women are weaker in their body than men, then it gets difficult of course. But it is up to each and every one, you might think it is fun and then it's no problem. But I think that gender roles play a major part in this, even if you don't think about it."

The participants said that women at the workplace were specialized in more work tasks than the men, which was described as adding to their workload and contributing to increased risk of vulnerability, tiredness and stress. Despite this the specialization was often expressed as a simple solution for everyone. Some participants questioned the essentialism of the gendered specialization and argued that it was a socialized pattern, whereas others viewed this as natural, as shown in the following discussion between two participants:

"- I mean, why do I have this talent? It might be because I followed that pattern.

- I think it's healthy that there are two gender roles, it's natural I think.

- But if you have done something many times then it gets easier and easier..."

Specialization was also considered as an explanation for the low number of men in elder care, as men generally were described as specialized in other things than caring. The caregivers' apprehension was that men avoided or left woman-dominated jobs because of social reprisals in terms of being looked down on by friends and acquaintances that did not see caring work as a proper job for a man. The participants in the focus groups said that they had chosen the job because of an interest in caring and because they liked their work. The men at the workplaces were described as exceptions to "men in general", who were described as more interested in high salaries than women. Despite the gendered patterns in the specialization, both in work tasks and in the labor market, many of the participants explained the differences in terms of personality and interests rather than gender.

In the category "women taking responsibility" the participants described the distribution of work tasks within the frames of the job description as spontaneous and based on different personalities. "We are different, we have different personalities and we, I mean maybe I am a person who wants to achieve and work overtime and take more responsibility than I need to." Women were often described as taking on more responsibility by seeing what needed to be done and doing it, but also as doing overambitious work at an unwarrantedly high pace. "I think that is common among women. You have a higher pace, and you assert yourself in some way by doing a lot, working a lot, in a different way."

Some caregivers divided the work tasks into basic work and extra work. The basic work was more evenly distributed among colleagues, whereas the extra work, such as taking care of flowers, changing curtains and curling hair, was unevenly distributed and usually performed by women. Men were often described as doing less or having a more relaxed attitude towards work. There was also an age difference, as the older women took more responsibility and to some extent also took the responsibility to tell their co-workers what to do. Some women were also described as taking individual responsibility for the skewed work division and tried to change their behavior to create an equal workload and a manageable workload for themselves.

"I have worked here for a long time and seen that it is those who are ambitious, even if you work full-time or part-time, it does not matter, it is those who are ambitious that work too much and get burnout, compared to those who only do their part and don't give a crap about the rest..."

Because the differences in work division and responsibility were not experienced as primarily gendered, but rather as influenced by personalities and interests, differences in workload were defined as a way of adapting the work situation to the individuals and not as gender inequality. The division of work tasks and responsibility was also dependent on differences in work capacities in the work group, related to health problems such as aches, pain or sleeping problems.

"You have a certain understanding because you know that she ... has an ache in her shoulder and you can see that today she seems to be worse and she has taken painkillers and then you don't want to put any extra load on that person."

However, it should be noted that this was expressed by the "helping" co-workers, and not the ones "being helped". The different work capacities resulted in an unequal work division which was accepted in order not to disturb the cooperation and the atmosphere. The task of caring for the elderly was described as taking priority over individual needs. The best solution for the work group was expected to be best for the individuals as well. This was expressed in statements such as "it does not matter who does something as long as it gets done" or "it is not possible to share everything equally, and as long as no one sees their part as a burden it is not a problem". Again, this situation seemed to be unfavorable for the women taking responsibility as they were the ones who ended up with a higher workload.

The category "managing family and health" shows that although cooperation and solidarity in the group was of great importance, work was often made a second priority after family life. There was a high level of acceptance for putting family first and staying home with sick children among the participants, which was justified by the low salaries in the professions. The participants argued that it was financially beneficial for the family that the parent with the lower salary stayed home with sick children. Both women and men with small children stayed home with sick children. However, women were described as taking the main responsibility for the home, which was seen as connected to women's higher levels of sick leave in general.

"I think that there are many things that a woman has to keep track of, maybe a sick relative or sick children. It depends, it is difficult to say because it depends on the situation at home. It's possible that women get more exhausted, I think."

Working part-time was considered as a solution to have time for the family but also to stay in good health despite the large workload. Working full-time was considered tough, and working part-time was a strategy to manage as "the body gets more rest". The private price of working part-time was lower salary and lower pension in the future. The caregivers also described how part-time work could be more intense than full-time, as their work was often done at the time of day when the workload was high. The part-time workers also had to make sure that they stayed updated on everything that was going on at work and sometimes they felt left out of the work group. The caregivers said that 75 percent working time was a critical limit, as working less included a lot of these negative consequences.

## Discussion

The caregivers in elder care advocated gender equality in principle, in terms of a less segregated labor market, equal salaries and equal workload. Gender equality was not spontaneously connected to health experiences for the participants. Gender inequalities were seen as something that should be managed at a higher level in the hierarchy, by managers, executives and perhaps politicians. This indicates that gender (in)equality was understood as part of a gender order of society, rather than a gender regime at the workplace level. Concurrently, the participants justified gender inequalities with individualism by focusing on differences in personalities and the advantages of specialization. The differences described as individual variations were often gendered. The focus on individualism seemed to make it possible to accept differences in responsibilities and workload between women and men. Therefore, the individual focus seemed to hide the gender structures of the workplace and also the importance that such gendered structures might have for health. The view of gender equality as a societal structure also positions gender equality at a distance from the individual, personal relations, and health experiences. Understanding gender equality issues as structural and health experiences as individual can possibly explain the disconnect for the participants between gender equality and health.

Concurrent presence of positive attitudes to equal opportunities and emphasis of practical obstacles related to individualism has been documented in earlier research on employment opportunities [5] and on the sharing of housework [52]. However, how this concurrency relates to health is underexplored. Despite the disconnect between gender equality at the workplace and own health, the focus on individual solutions legitimated gender inequalities in workload and responsibilities which in turn could be related to health experiences. Taking on responsibility was seen as a personality trait that many women had which added to women's workload. The women who took responsibility and put their own need second to the needs of their patients were necessary for the workplace to function, but the negative consequences of work overload and stress were viewed as individual problems. To regard individual factors as responsible for gendered work division has been described as an intertwining of gender identities and workplace organizing practices [53]. It is problematic when caring or attentiveness is viewed as an expression of personality in this way, as it might be part of a survival mechanism for those in subordinate social positions [29]. Taking on additional responsibilities to protect patients from insufficient health care systems has previously been recognized as a health risk for nurses and home care workers because of increased workload [40]. When the gender identities and workplace organizing practices are intertwined there is a risk of constructing a caring femininity in which personal needs are neglected in order to care for others. This type of femininity might also be connected to negative health experiences of stress and burnout, but more research is needed on this topic. In a setting with inadequate staffing and insufficient resources the caregivers might feel forced to take on an unhealthy workload to meet the demands. Taking responsibility could, however, also be related to work satisfaction, which may protect against some of the negative health consequences of the extra workload. When justifying gender inequalities the participants seemed to make use of a gender difference perspective, whereas a sameness perspective was used when advocating gender equality.

Bringing more men into the occupation was seen as a solution to problems of low salaries and low status. Men were also expected to bring a different banter to the workplace. This type of expectations on men in woman-dominated work have been discussed as problematic in masculinity research, as they put men in a position where they are first and foremost male and professionals in second place [54]. The emphasis on differences between women and men may make it difficult for the men to engage fully in work tasks characterized as feminine and might therefore create gender inequalities in the division of work and responsibilities at the workplace. Therefore, a gender sameness perspective is needed to promote gender equality at the workplace, where men in caregiving professions can be appreciated as caregivers and not as contributing something different and "masculine" [55]. If the qualities of a good caregiver are intertwined with femininity this might cause problems for men who enter the profession and result in a gendered division of work tasks. However, the men in our study were described as interested in caring and thereby different from "men in general", who were described as making economic career choices. There is therefore a possibility that the view of men as contributing something different is changed once they enter the workplace. Expanding masculinity to include caring has also been suggested to be health-promoting, as increased responsibility for others might bring less risky behaviors [56].

Another expression of individualism among our participants was the solution of working part-time. Parttime work was seen as enabling the caregivers to stay healthy despite a heavy workload and also to achieve flexibility at work and create a possibility to accommodate work to family life. This indicated that work-life balance in this paper, just as in other studies, is perceived to be a personal rather than a structural issue, dealt with by using individual strategies [57]. However, working part-time was mostly mentioned as a solution for the women, and this individualism was therefore mainly related to fulfilling gender norms and meeting the obligation for women to care for children and old relatives. It is important to acknowledge that working part-time as a strategy to diminish workload only is efficient if it is not accompanied by added responsibilities of unpaid work. The individual solution of working part-time also has negative consequences for income and career opportunities. Similar patterns of adapting work to fulfill gender norms has previously been documented among self-employed women and men [58], along with the obligation for women to care for family [59]. In our setting the focus on individualism in a work perspective is ambivalent as it may mean adapting to the needs of the family, or gender norms rather than individual health needs.

A healthy-setting approach that focuses on changing the social and/or physical environment and, through this, individual behavior [60] might be an effective policy measure to move away from individual solutions. To achieve social change the gendered patterns of the socalled individual choices need to be scrutinized both in relation to the work tasks and in relation to working time and family responsibility. Variation between all kinds of work tasks for women and men in the same profession needs to be encouraged.

### Methodological Considerations

We ensured the credibility of the study throughout the data collection and data analysis in various ways. In the focus group discussions the moderator and assistant moderator worked actively to create an open, friendly and accepting discussion. The moderator emphasized that everyone's experiences and thoughts were valuable. In the analysis process the researchers repeatedly returned to the transcripts to confirm the interpretations. One researcher conducted the coding process in discussions with the other researchers to ensure a high level of coding consistency [61]. We deemed the material from the focus groups to be rich, presenting a variety of experiences, views and opinions and therefore sufficient for the intended analysis.

Our intended focus in the study was the relation between gender equality at work and health experiences. However, in the focus group discussions the moderator and assistant moderator found it difficult to get the participants to talk about the connection between gender equality and health. The discussions tended to focus on either gender (in)equality issues or health. The aim of the paper has therefore been formulated to grasp the meaning of gender equality at the workplace and discuss what this may mean for the workers' health experiences. This drift in the research question might have been avoided if we had applied an emergent design where we could have adjusted the questions to more precisely cover our original research question. However, the problems encountered might also be related to a general difficulty for individuals to see their place in a gendered organization. Other researchers have found that gender in(equality) is often described by study participants as an organizing principle at other workplaces and in society at large, but not at one's own workplace [37]. Such a position, which is also present in our results, makes it difficult to discuss one's own health experiences related to a gender inequality that is not perceived as such.

In this study we were interested in the general view of gender equality at a workplace in elder care with a majority of women employees. Each participant in the group is affected by group dynamics, which makes it inappropriate for comparisons between participants within one group on individual characteristics such as gender or profession if segmentation is not used [49]. Also, due to ethical standards the few men in our study could have been recognized if the gender of the participants had been added. We did however analyze the participants' views of women and men that were expressed in the focus groups.

## Conclusions

The caregivers in elder care advocated gender equality in principle but did not see connections between gender equality at work and their own health experiences. Concurrently, they justified gender inequalities with individualism, resulting in a gendered work division which may result in health inequalities between women and men. The simultaneous confirmation of a gender equality ideal and justification of gender inequality blurs the possibility to see the association between gender inequality and health experiences of women and men. The justifications of gender inequality must therefore be at the center of our attention to enable gender equality in health. Our results indicate that a perspective stressing individualism and gender difference on the workplace level plays an important part in the justification of gender inequalities, and that individual solutions might have negative structural consequences for health.

## Competing interests

The authors declare that they have no competing interests.

## Authors' contributions

All authors participated in the design of the study. SE and LA performed the data collection as moderator (SE) and assisting moderator (LA) of the focus group discussions. All authors contributed to the analysis of the data. SE drafted the manuscript and AH and LA critically revised the intellectual content of the manuscript. All authors read and approved the final manuscript.
